# Field safety and efficacy of a unique live virus vaccine for controlling avian encephalomyelitis and fowlpox in poultry

**DOI:** 10.14202/vetworld.2019.1291-1298

**Published:** 2019-08-23

**Authors:** Girish Sarma, Barry A. Kersting, Gary Spina

**Affiliations:** Hygieia Biological Laboratories, P.O. Box 8300, Woodland, California 95776, USA

**Keywords:** avian encephalomyelitis, efficacy, field safety, fowlpox, live virus vaccine, pigeon pox, protection

## Abstract

**Background and Aim:**

Infection of commercial poultry with avian encephalomyelitis (AE) and fowlpox (FP) virus causes heavy economic loss in endemic areas. Although vaccines are routinely used to control these two diseases, the problem still persists almost all over the world. This study aimed to evaluate safety and efficacy of a unique AE + FP + pigeon pox (PP) live virus vaccine in layer-type chickens under both laboratory and field conditions.

**Materials and Methods:**

The study was conducted using 289 specific-pathogen-free (SPF) chickens under the laboratory conditions and 185,648 commercial layer-type chickens under field conditions. In two consecutive laboratory trials, 8-week-old SPF chickens were vaccinated with the AE + FP + PP live virus vaccine through wing web route and challenged against virulent strains of FP and AE viruses at 3-week post-vaccination (WPV). Challenged chickens were observed for disease protection for 10-21 days. For field safety trials, commercial layer-type chickens in three different geographical areas in the USA were vaccinated with the AE + FP + PP vaccine and observed daily up to 21 days for vaccine “take,” adverse reactions, and mortality.

**Results:**

The vaccine was found safe and efficacious under both laboratory and field conditions. Vaccine “take” and protection against FP challenge were 100%. Average protection against AE challenge was 97%. Mean AE enzyme-linked immunosorbent assay (ELISA) antibody titer in the field vaccinated chickens was >1200 at 10 WPV. Average daily post-vaccination mortality in the field vaccinated chickens was 0.04%. So far, more than 400 million chickens in the USA have been vaccinated with this vaccine. No vaccine-associated adverse reactions, other safety issues, or immunity breakdown cases in the vaccinated flocks due to field virus infection have been reported.

**Conclusion:**

This unique vaccine containing AE, FP, and PP viruses in a single preparation was found to be safe and efficacious in controlling the diseases caused by the virulent field strains of AE and FP. Besides being safe and efficacious, this vaccine also offered distinct advantages over the traditional vaccination practices in controlling these two diseases in poultry.

## Introduction

Avian encephalomyelitis (AE) and fowlpox (FP) are two of the common viral diseases of poultry worldwide. Although AE virus primarily infects chickens, the virus has also been isolated from other avian species including turkeys and quail [1-3]. The avipoxviruses, on the other hand, have been isolated from numerous avian species including chickens, turkeys, canaries, flamingo, and others [4-12].

In natural outbreaks of FP in chicken, the primary lesions (scabs or dry pox) are usually seen in the injured unfeathered areas of the skin around the head and mouth such as comb, wattle, ear lobes, and eyes [5,10,12]. These primary lesions may spread to other areas of the skin forming the secondary lesions. Infection of susceptible chickens with more virulent field viruses often results in the development of diphtheritic lesions (wet pox) in the mucous membrane of the oral cavity and the upper respiratory tract, especially the larynx and trachea [5,10,12]. Wet pox induces higher mortality in infected flocks resulting in significant economic loss, drop in egg production in layers, and reduced growth rate in the broilers. Concurrent infection with other infectious agents may increase the severity and course of the disease in the infected birds [8]. Integration of reticuloendotheliosis virus genome sequences of various lengths in the FP virus genome has been reported to enhance the pathogenicity of the virus resulting in the emergence of the very pathogenic or variant strains of the virus [13]. The AE virus infection in laying and breeding flocks causes a marked drop in egg production, decrease in egg hatchability, and high mortality in young infected chicks [1-3]. To control these two diseases, poultry producers routinely vaccinate their flocks with AE and FP live virus vaccines [14-19]. However, in spite of vaccination, immunity breakdown cases due to very virulent or variant field strains of the virus have been often reported in the FP vaccinated flocks [17,18]. Studies on the immunity breakdown cases revealed that immunity induced by FP vaccine alone was not sufficient enough to protect against the virulent field strains. Under such situation, vaccinating chickens with a mixture of FP and pigeon pox (PP) vaccines appeared to provide better protection against the virulent field strains of the virus [14,20]. The improved protection observed following administration of the mixed FP and PP vaccines in chickens was thought to be due to generation of the cross-protective immune response against the other [20].

In the pox endemic areas, poultry producers vaccinate their flocks with a mixture of both FP and PP vaccines to obtain a greater spectrum of protection against the disease. However, until recently, the usual vaccination practice was to mix various FP containing products with PP vaccine in the field and administer the mixed product to the chickens by wing web (WW) route. However, mixing of separate products in the field has many disadvantages including preparation error, increased cost, longer time requirement for vaccine rehydration and mixing and eventual loss of virus titer, inconsistency in vaccine potency, and product contamination during preparation and processing. Availability of a safe and efficacious vaccine containing all of these three viruses in a single preparation would, therefore, be unique in its nature, user-friendly, cost-effective, and eventually meet the long awaiting needs of the poultry industry.

This study aimed to evaluate the safety and efficacy of a lyophilized live virus vaccine containing all of the three viruses (AE, FP, and PP) in a single preparation. Since this combination is unique and no other similarly combined product is currently available from other vaccine manufacturers in the poultry vaccine market elsewhere, a critical evaluation of the product in chickens was done following the approved guidelines and protocols of the veterinary biologic products licensing authority in the USA before marketing the vaccine for commercial use. The results obtained from the studies conducted in layer-type chickens under both laboratory and field conditions are presented in this report.

## Materials and Methods

### Ethical approval

No ethical approval was needed for this research.

### Chickens

For safety and efficacy evaluation of the vaccine under laboratory conditions, two hundred and eighty-nine 8-week-old specific-pathogen-free (SPF) chickens (Source: Valo Biomedia, Adel, IA) were used. Two consecutive vaccination/challenge trials (Trial 1 and Trial 2) were conducted to validate the reproducibility of the test results (Table-1). For Trial 1, 149 chickens were used. Another 140 chickens of the same breed, age and source were used for Trial 2. In each trial, chickens were wing banded and divided into five groups. Chickens in Group 1 and 2 were vaccinated with the AE + FP + PP vaccine and challenged against AE and FP, respectively. Chickens in Groups 3 and 4 were the positive controls (unvaccinated challenged) for AE and FP challenge, respectively. Chickens in Group 5 were kept as a negative control (unvaccinated unchallenged).

**Table 1:** Protection in AE+FP+PP vaccinated chickens against challenge with virulent strains of AE and FP.

Trial number	Treatment groups	Treatment ID	Number of chickens	% Post- vaccination “take” for FP	Vaccine induced mortality/adverse reaction	Number of chickens challenged		% Protection	
	
						FP	AE	FP	AE
1	1	Vaccinated	32	100	0/32	32	-	100	-
	2	Vaccinated	32	100	0/32	-	30*	-	93
	3	Positive control	32	n/a	n/a	32	-	0	-
	4	Positive control	32	n/a	n/a	-	31*	-	0
	5	Negative control	21	n/a	n/a	n/a	n/a	n/a	n/a
2	1	Vaccinated	30	100	0/30	30	-	100	-
	2	Vaccinated	30	100	0/30	-	30	-	100
	3	Positive control	30	n/a	n/a	30	-	0	-
	4	Positive control	30	n/a	n/a	-	30	-	0
	5	Negative control	20	n/a	n/a	n/a	n/a	n/a	n/a
Trials 1 and 2 combined		Vaccinated	124	100	0/124	62	60	100	97
		Positive control	124	n/a	n/a	62	61	0†	0†
		Negative control	41‡	n/a	n/a	n/a	n/a	n/a	n/a

* Two vaccinated and one positive control bird died on the day of AE challenge due to mechanical injury. †All AE and FP positive controls in both trials became positive for AE and FP clinical signs respectively after challenge. ‡41/41 negative controls in both trials remained healthy and free of AE or FP clinical signs and/or mortality during the period of observation. n/a=Not applicable

For safety evaluation of the vaccine under field conditions, commercial layer-type chickens aged 8-12 weeks and located in three different geographical areas in the USA (Site 1-3) were used (Table-2). At each trial site, two groups of chickens were used for evaluating the safety of the test product. Chickens in the test group were vaccinated with the AE + FP + PP vaccine containing AE, FP, and PP viruses in a single preparation. The total number of chickens vaccinated with the AE + FP + PP vaccine in these three trial sites was 185,648. For comparing the results of the AE + FP + PP vaccine, almost equal number of chickens of the same breed, age and source were used as controls. The total number of control chickens used in all of the three trial sites was 184,060. The control chickens in each of the three field trial sites were also vaccinated, but they were vaccinated following the normal vaccination program of the participating farms which included all necessary live virus vaccinations including AE, FP, PP, infectious laryngotracheitis (ILT), and other live virus vaccines obtained through different manufacturers.

**Table 2:** Post-vaccination pox “take” in the field vaccinated chickens.

Trial sites	Treatment groups	Number of chickens vaccinated	Breed	Age at vaccination	Vaccines used	Pox vaccine “take” (%)*	

						Positive	Negative
Site 1	Vaccinated	74,684	LSL	8 Weeks old	AE+FP+PP	100	0
	Controls	75,705	LSL	8 Weeks old	AE+FP & PP Mix	97	3
Site 2	Vaccinated	39,766	Cobb	10 Weeks old	AE+FP+PP	100	0
	Controls	40,147	Cobb	10 Weeks old	AE+FP & PP Mix	100	0
Site 3	Vaccinated	71,198^†^	Hy-W-36	11 Weeks old	AE+FP+PP	100	0
	Controls	68,208^‡^	Hy-W-36	11 Weeks old	AE+FP vectored ILT	61	39

* Results of examining 100 randomly selected chickens from each treatment group at each trial site at 6 to 8 DPV.

† All chickens in this AE+FP+PP vaccinated flock were also simultaneously vaccinated with ILT vaccine, modified live virus.

‡ The control chickens in this trial site were vaccinated with the AE + fowlpox vectored ILT vaccine.

### Vaccines

Lyophilized vaccines containing chicken embryo propagated live AE, FP, and PP viruses in a single preparation were used in this study. The virus strains used in the vaccine were same as other commercially available vaccines in the US poultry vaccine market. Each vial of vaccine containing 1000 doses was supplied with a vial of sterile diluent for vaccine reconstitution and one double-needle applicator for vaccine administration through the WW route. The sterile diluent contained blue dye to facilitate examination for “take” following vaccine administration. Before use, each vial of vaccine was rehydrated with 10 ml sterile diluent. Each of the three viral fractions in the vaccines (AE, FP, and PP) had a titer of ≥10^4.6^ EID_50_/ml at formulation and passed all standard quality control requirements of the vaccine manufacture. For field safety trial, the vaccine was supplied to the trial participants along with sterile diluent, double-needle applicators, and a detail instruction for use circular.

### Method of vaccination

For laboratory Trials 1 and 2, the vaccine was administered to the SPF chickens through the WW route using a double-needle stab applicator. Each double-needle stab delivered one dose in 0.01 ml of the reconstituted vaccine in the midportion of the web of one wing. Before use, each vial of vaccine was rehydrated with 10 ml sterile diluent. The positive and negative control chickens for Trials 1 and 2 were not vaccinated but inoculated with 0.01 ml of the sterile diluent (placebo) only in one of their WWs using a double-needle applicator.

In all of the three field trial sites, the vaccine was administered to the chickens by the vaccination crew of the field trial participants. The method of vaccine rehydration and administration in all field trial sites was the same as described before.

### Post-vaccination observation and evaluation of pox vaccine “take” under laboratory conditions

Following vaccination, all vaccinated and control chickens of laboratory Trials 1 and 2 were housed in separate housing units and managed under identical climatic conditions. Food and water were provided to all the chickens *ad libitum* during the entire period of observation. All chickens (vaccinated and controls) were observed daily up to 3-week post-vaccination (WPV) for vaccine-associated mortality, adverse reactions, development of clinical signs of virulent pox virus infection such as appearance of pox lesions on the comb, wattle, eyelids, and other non-feathered areas of the body, wet pox or diphtheritic lesions in the mucous membrane of the oral cavity, and grossly noticeable physical appearance and health status. The adverse reactions included any bird appearing sick and debilitated, lethargic and unwilling to move, and reduced feed and water consumption. All vaccinated and control chickens were also examined at 6-day post-vaccination (DPV) for pox vaccine “take” at the site of vaccine administration. A “take” is a nodular swelling or a scab which develops within 4-8 DPV at the site of vaccination in the WW and is commonly used to evaluate successful poxvirus vaccination. Depending on the size of the nodular swelling, a “take” is described as readily palpable or large and barely palpable or small. The size of the readily palpable or large “takes” measured approximately 6-8 mm in diameter and the size of the small “takes” measured 3 mm or less in diameter.

All vaccinated as well as control chickens were also observed for the development of clinical signs of AE including ataxia, circling, depression, paralysis, sudden death, tremors, or torticollis. All vaccinated and control chickens were housed separately until challenged. After challenge, the vaccinated and control chickens were comingled. The AE challenged chickens were comingled separately from the pox challenged chickens.

### Post-vaccination observation and evaluation of pox vaccine “take” under field conditions

Under field conditions, both vaccinated and control chickens in all three trial sites were observed daily for vaccine-induced adverse reactions, daily mortality, and development of clinical signs of virulent AE or FP virus infection for a period up to 3 weeks. At 6-8 DPV, 100 randomly selected chickens from each treatment group in each trial site were also examined for pox vaccine “take” at the site of vaccine administration. In each trial site, all pre- and post-vaccination observations including post-vaccination adverse reactions, daily mortality, and examination for pox “take” were done by the designated farms’ personnel. Seroconversion against AE was assessed by testing the post-vaccination serum samples by enzyme-linked immunosorbent assay (ELISA) at different intervals after vaccination. The participating farms collected and sent the serum samples to a state disease diagnostic laboratory for serological evaluation.

### Method of challenge

At 3 WPV, all vaccinated and positive control chickens in laboratory Trials 1 and 2 were challenged against AE and FP. Chickens in Groups 1 and 3 in each trial were challenged against FP using a standard challenge strain of FP virus received from the USDA. The FP challenge virus was diluted 1:10 with Tryptose Phosphate Broth (TPB) and administered to all vaccinated and positive control chickens by WW stab method using a double-needle applicator. The wing that was opposite to the vaccinated wing was used for FP challenge virus administration. The challenge dose was 0.01 ml for each chicken. Chickens in Groups 2 and 4 in each trial were challenged against AE using a virulent strain of AE challenge virus also received from the USDA. The AE challenge virus was diluted 1:10 in TPB and 0.05 ml of it was administered to each chicken by the intracerebral route.

The FP challenged chickens were thoroughly examined for the development of pox lesions for up to 10-day post-challenge (DPC). Each chicken was checked for the development of primary pox lesion at the site of challenge virus administration as well as for secondary lesions in all non-feathered areas of the body including the comb, wattles, and eyelids. Chickens were also examined for the development of diphtheritic lesions in the buccal mucosa, cloaca, and vent. The absence of any grossly detectable pox-specific lesion indicated protection against pox virus challenge. Any chicken showing lesions or clinical signs of FP virus infection and/or mortality was considered positive or unprotected. The absence of any FP virus-induced clinical signs and/or mortality indicated protection against FP challenge.

The AE challenged chickens were observed for 21 DPC for AE virus-induced clinical signs such as ataxia, circling, depression, paralysis, sudden death, tremors, or torticollis. Any chicken showing clinical signs of AE virus infection and/or mortality was considered positive or unprotected. The absence of any AE virus-induced clinical signs and/or mortality indicated protection against AE challenge.

In this study, challenge evaluation of the AE and FP immunity was done only for the laboratory Trials 1 and 2. The field vaccinated birds were not brought to the laboratory for challenge work. Instead, all field vaccinated chickens were observed for disease protection against natural challenge with the virulent field strains of the viruses.

## Results

### Safety of the vaccine under laboratory conditions

The results of post-vaccination safety observation in the vaccinated chickens of Trials 1 and 2 are presented in Table-1. There was no vaccine-associated mortality or adverse reactions in any of the vaccinated chickens in these trials. All vaccinated chickens remained healthy and active as the unvaccinated controls. Physical examination of each chickens showed no signs of virulent AE or FP virus infection or lesions induced by these viruses in any of the vaccinated chickens except the development of pox “take” at the site of vaccine administration (WW). When examined at 6 DPV, readily palpable pox “takes” were seen in 100% of the vaccinated chickens. These nodular swellings at the site of vaccine administration completely healed and disappeared in 2-3 weeks.

### Efficacy of the vaccine under laboratory conditions

The post-vaccination challenge results of the laboratory Trials 1 and 2 revealed 100% protection against FP challenge in both trials (Table-1). Protection against AE challenge was 93% in Trial 1 and 100% in Trial 2 (average 97%). Protection in the positive control chickens was 0% against both challenge viruses. The negative control chickens remained free of AE or FP clinical signs during the entire period of this study.

### Safety of the vaccine under field conditions

**Figure-1:**
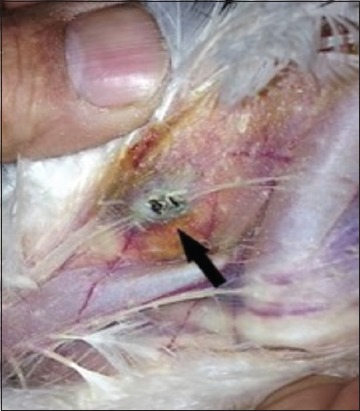
Pox vaccine “take” at 6-8 days post-vaccination in the avian encephalomyelitis + fowlpox + pigeon pox field vaccinated chickens.

The results of field safety trials conducted in three different geographical areas in the USA are summarized in Tables-2 and 3. Following vaccination with the AE + FP + PP vaccine, 100% of the vaccinated chickens in all of the three trial sites developed readily palpable pox “takes” at the site of vaccine administration (Figure-1 and Table-2). In the control flocks, although, total pox “takes” varied from 61% to 100%, on an average 20% of these “takes” were quite small and difficult to palpate. No adverse reactions such as abscess formation or extensive tissue damage at the site of vaccine administration were seen in any of the vaccinated chickens.

**Table 3:** Post-vaccination mortality in the AE+FP+PP vaccinated flocks compared to the control flocks.

Trial sites	Treatment groups	Number of chickens vaccinated	Average daily mortality (%)					Vaccine induced adverse reactions

			Pre-vaccination (average of 3 days just before vaccination)	Post-vaccination				

				Average of 1^st^ WPV	Average of 2^nd^ WPV	Average of 3^rd^ WPV	Average of all 3 WPV	
Site 1	Vaccinated	74,684	0.02	0.01	0.01	0.02	0.01	None
	Control	75,705	0.02	0.01	0.01	0.01	0.01	None
Site 2	Vaccinated	39,766	0.02	0.02	0.04	0.03	0.03	None
	Control	40,147	0.01	0.02	0.01	0.03	0.02	None
Site 3	Vaccinated	71,198	0.01	0.03	0.04	0.15*	0.07*	None
	Control	68,208	0.01	0.02	0.01	0.01	0.01	None
Total of three sites	Vaccinated	185,648	0.02	0.02	0.03	0.07	0.04	None
	Control	184,060	0.01	0.02	0.01	0.02	0.02	None

* Slight increase in mortality at 3WPV was due to the post-vaccination reaction associated with the simultaneous administration of ILT modified live virus vaccine to the AE+FP+PP vaccinated birds by eye drop method.

The results of pre- and post-vaccination average daily mortality records in the AE + FP + PP vaccinated and control chickens in all of the three field trial sites are shown in Table-3. The average daily pre-vaccination mortality in the AE + FP + PP vaccinated and control flocks in all of the three trial sites ranged from 0.01% to 0.02%. The post-vaccination average daily mortality (average of 3 WPV) in the AE + FP + PP vaccinated chickens in these three trial sites ranged from 0.01% to 0.07%. In the control flocks, the average daily mortality (average of 3 weeks) ranged from 0.01% to 0.02%. The overall post-vaccination average daily mortality during the 3 WPV periods in all of the three trial sites was 0.04% in the vaccinated flocks and 0.02% in the control flocks.

### Efficacy of the vaccine under field conditions

**Figure-2:**
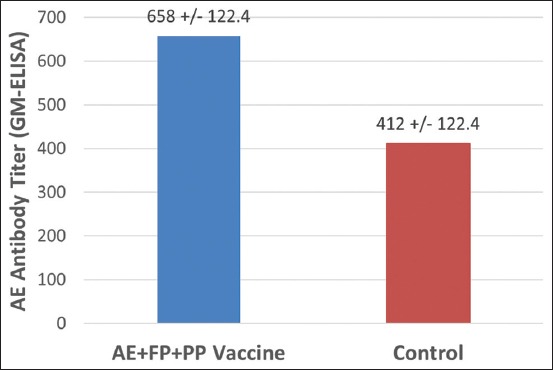
Avian encephalomyelitis (AE) antibody titer in the AE + fowlpox + pigeon pox vaccinated chickens compared to the control flocks. Number of serum samples tested = 11.

**Figure-3:**
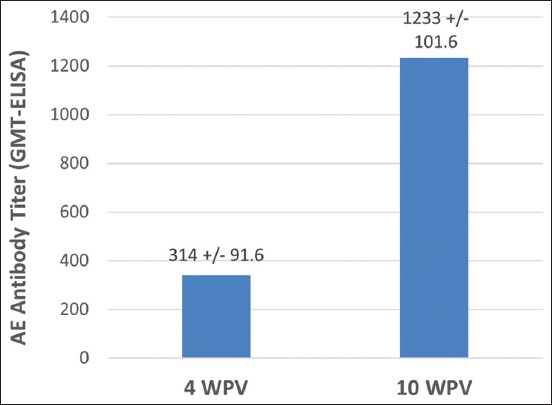
Avian encephalomyelitis antibody titer at 4 and 10 weeks post-vaccination in the field vaccinated chickens. Number of serum sample tested = 20.

Although no challenge studies were conducted with the field vaccinated chickens, overall field observations revealed no AE or FP outbreaks in the vaccinated flocks. The AE + FP + PP vaccinated chickens remained free of the clinical signs caused by field exposure with the virulent field strains of AE or FP viruses. The results of AE seroconversion study in the field vaccinated chickens are shown in Figures-2 and 3. At 6 WPV, the geometric mean ELISA antibody titer against AE in the AE + FP + PP field vaccinated chickens was 658±122.4, whereas the titer in the control chickens was 412±122.4 (Figure-2). The AE ELISA antibody titer gradually increased the following vaccination. Titers at 10 WPV were almost 4-fold higher than at 4 WPV (Figure-3).

More than 400 million chickens have been vaccinated so far in the USA using this vaccine. No vaccine-associated adverse reactions or safety issues have been reported by its users. All vaccinated flocks did well with respect to their protection against AE and FP caused by the virulent field strains of the viruses. No immunity breakdown cases due to AE or FP field virus infections have been reported in the AE + FP + PP vaccinated chickens. Under field conditions, the vaccine consistently produced distinct, readily palpable pox “takes” in 100% of the vaccinated chickens. Besides being safe and efficacious, this vaccine offered obvious advantages over the traditional vaccination practices in controlling these two diseases in poultry.

## Discussion

The results of this study indicated that this AE + FP + PP vaccine was safe and efficacious under both controlled conditions in the laboratory as well as under field conditions. In both laboratory trials (Trials 1 and 2), the vaccine was found to be safe for WW administration in 8-week-old chickens. This was evidenced from the absence of any vaccine-induced adverse reaction, leading to increased mortality, absence of wet pox or secondary pox lesions at the non-inoculated sites, and absence of AEV-induced clinical signs and/or mortality during the 3 WPV observation period (Table-1). Since the vaccine was administered through the WW route (not orally) and the virus strains in the vaccine have been in use in poultry industry for more than 50 years without any apparent safety concern, no shed-spread studies were conducted in the vaccinated chickens. Instead, we performed reversion to virulence studies with the vaccine strains by giving five consecutive bird-to-bird passages and observing for the development of clinical signs of AE and FP virus infection in the virus inoculated chickens at each passage. No reversion to virulence or any other safety issues were observed in any of the vaccinated chickens (Unpublished data). Furthermore, the safety of the vaccine viruses in the susceptible chickens was also confirmed by experimentally administering the vaccine using a much higher dose (≥10 times of the recommended dose) and then observing the vaccinated chickens for any unfavorable reactions, mortality, or development of specific clinical signs of the disease induced by the vaccine viruses. Administration of vaccine viruses at a much higher dose did not adversely affect the safety of the vaccine (Unpublished data).

The vaccine was found to be safe under field conditions also. This was demonstrated by the absence of any post-vaccination adverse reaction and/or a significant increase in daily mortality attributable to the vaccine in the vaccinated chickens in all of the three field trial sites (Table-3). Out of a total of 185,648 chickens vaccinated with the AE + FP + PP vaccine, the average daily mortality up to 3 WPV was only 0.04%. Although a slight increase in average daily mortality was observed in the vaccinated chickens in one of the three field trial sites, the values were still well within the acceptable range of the participating farms. Under normal field circumstances in the large egg-laying flocks with modern disease biosecurity system and husbandry conditions, average daily mortality of up to 1.0% is considered quite normal [21]. In this study, the slight increase in daily mortality in trial site 3 was not related to the administration of the AE + FP + PP vaccine. In fact, this transient increase in mortality was due to simultaneous administration of other live virus vaccines to the AE + FP + PP vaccinated chickens. The AE + FP + PP vaccinated chickens at this site were simultaneously vaccinated with modified live ILT virus vaccine by the eye drop method. The increase in mortality at 3 WPV was determined to be due to the vaccination reaction caused by the modified live ILT vaccine administration. Chickens in the control flock in this site were not vaccinated with the live modified ILT virus vaccine. Instead, they were vaccinated with the FP vectored – ILT vaccine which does not induce ILT virus-associated post-vaccination reaction and mortality. Increase in mortality following modified ILT live virus vaccine administration is a well-recognized problem [22,23].

The AE + FP + PP vaccine induced very good, readily palpable pox vaccine “take” in all vaccinated chickens both under the laboratory and field conditions (Tables-1 and 2). However, the pox vaccine “takes” in the control flocks in the field was minimal, smaller in size, and difficult to palpate in many cases. The “takes” in the control flocks were less than half in size of the AE + FP + PP vaccinated flocks. Checking for post-vaccination “take” is considered to be one of the best methods of evaluating pox immunity in vaccinated chickens. An efficacious vaccine, when administered properly, is expected to show “takes” in 99%-100% of the vaccinated chickens. In recent years, low pox vaccine “take” has been frequently observed in the FP vaccinated chickens in the field. The absence of pox “take” in the FP vaccinated chickens could be due to several factors including faulty vaccination, presence of pox antibody from previous field exposure or vaccination, and low virus titer in the vaccine [24,25]. Insufficient virus titer in the vaccine and/or presence of pox antibodies in the chickens at the time of vaccination may adversely affect pox “take” development, thus producing small or difficult to palpate “takes” in the vaccinated chickens. The duration of immunity in such chickens remains questionable.

The results of the vaccination/challenge studies under laboratory conditions indicated that both AE and pox fractions of the vaccine were efficacious and protected the vaccinated chickens against challenge with virulent strains of the AE and FP viruses (Table-1). While protection against FP challenge was 100%, average protection against AE challenge was 97%. This could be due to the route of administration of the AE challenge virus. Although for AE virus, the natural route of infection is by the oral route, we challenged the chickens through the intracerebral route. It was also worth noting that mixing of AE, FP, and PP viruses in a single preparation did not adversely affect or interfere with the immunogenic potential of any of the components of the vaccine preparation. A parallel vaccination/challenge trial conducted by the authors using the monovalent AE, FP, and PP vaccines in 8-week-old SPF chickens following similar vaccination and challenge protocol revealed 100% protection against FP challenge and 96% protection against AE challenge (Unpublished data).

In the present study, challenge evaluation of the post-vaccination immunity was done only for the laboratory trials under controlled conditions. Field vaccinated chickens were not brought to the laboratory for challenging against either AE or FP viruses. Instead, chickens vaccinated under the field conditions were observed for disease protection against natural challenge with the field strains of these viruses. All vaccinated flocks were routinely observed for immunity breakdown cases such as occurrence of AE or FP outbreaks in the vaccinated birds. No immunity breakdown cases were reported in any of the field vaccinated chickens. Besides this, randomly collected post-vaccination serum samples from the field vaccinated chickens were also tested for AE seroconversion to test the efficacy of the vaccine against AE. The results of the post-vaccination serology indicated that the AE + FP + PP vaccine induced very good seroconversion against AE (Figures-2 and 3). The ELISA antibody titer gradually increased from 4 WPV to 10 WPV (maximum period tested). Unlike some other viral vaccines, AE seroconversion may not be complete within 3-5 WPV. Furthermore, the level of AE antibody achieved in the early weeks following vaccination may depend on several other factors including bird type, level of field virus exposure, maternal antibody, and route of vaccine administration [16]. Thus, testing of serum samples 3-5 WPV may not accurately reflect the immune status of the flock. More accurate results are obtained when the flock is first tested at 7-10 WPV and then retested before the start of lay. AE ELISA antibody titer of ≥1:400 has been shown to provide 100% of progeny embryos resistance to viral challenge [15].

## Conclusion

This freeze-dried, live virus vaccine containing AE, FP, and PP viruses in a single preparation was found to be safe and efficacious when tested in layer-type chickens both under the controlled conditions in the laboratory and under the field conditions in three different geographical areas in the USA. The vaccine provided complete protection against the diseases caused by virulent strains of the viruses. More than 400 million chickens in the USA have been vaccinated so far with this vaccine. No vaccine-associated adverse reactions, other safety issues, or immunity breakdown cases due to field virus infection have been reported in the vaccinated chickens. Besides being safe and efficacious, the vaccine offers obvious advantages over the traditional vaccination practices where PP vaccines are mixed with the AE and FP vaccines in the field before vaccination. Mixing of separate vaccines in the field has several disadvantages such as increased vaccination cost, possible errors in vaccine rehydration and mixing, and loss of vaccine potency due to increased time required for vaccine preparation. This pre-mixed and pre-standardized product containing AE, FP, and PP viruses in a single preparation eliminates all of these disadvantages and stands out as a unique user-friendly product for the poultry industry.

## Authors’ Contributions

GS contributed by providing the original idea on this unique product, designing the entire study for complete evaluation of the vaccine, its formulation, performing all vaccinations and challenge works in the laboratory, evaluating the challenge results, and preparing the manuscript. BAK helped in conducting the experiments in chickens under laboratory conditions. GSp contributed by conducting the field trial studies in commercial chickens. All authors read and approved the final manuscript.
